# *Marchantia polymorpha* L. ethanol extract induces apoptosis in hepatocellular carcinoma cells via intrinsic- and endoplasmic reticulum stress-associated pathways

**DOI:** 10.1186/s13020-021-00504-4

**Published:** 2021-09-28

**Authors:** Fangfang Zhou, Adila Aipire, Lijie Xia, Xierenguli Halike, Pengfei Yuan, Mamtimin Sulayman, Weilan Wang, Jinyao Li

**Affiliations:** Xinjiang Key Laboratory of Biological Resources and Genetic Engineering, College of Life Science and Technology, Xinjiang University, Urumqi, 830046 China

**Keywords:** *Marchantia polymorpha* L., Hepatocellular carcinoma, Apoptosis, Signaling pathway, Tumor mouse model

## Abstract

**Background:**

*Marchantia polymorpha* L. is a kind of Chinese herbal medicine and has various biological activities including antioxidant and antifungal. However, it is not clear about the antitumor effect and mechanism of *M. polymorpha*. We prepared *M. polymorpha* ethanol extract (MPEE) and investigated its antitumor effect on hepatocellular carcinoma cells both in vitro and in vivo.

**Methods:**

The viability of hepatocellular carcinoma cells was detected by MTT assay. The distribution of cell cycle was analyzed by propidium iodide (PI) staining. The morphology of nuclei was observed by Hoechst 33258 staining. Apoptosis was detected by Annexin V/PI staining. JC-1 fluorescent probe and DCFH-DA were used to detect the mitochondrial membrane potential (ΔψM) and the level of reactive oxygen species (ROS), respectively. Caspase inhibitors were used to test the function of caspase in the induction of apoptosis. Quantitative real time polymerase chain reaction (qRT-PCR) and Western blot were used to evaluate the levels of mRNA and protein, respectively. Differentially expressed genes and signaling pathways were identified by transcriptome analysis. The H22 tumor mouse model was used to detect the antitumor effect of the extract.

**Results:**

MPEE significantly suppressed the migration and growth of BEL-7404, HepG2 and H22 cells in a dose- and time-dependent manner through induction of apoptosis characterized by chromosomal condensation and cell cycle arrest at G0/G1 and G2/M phases. MPEE induced mitochondria-dependent apoptosis via upregulation of Bax and downregulation of Bcl-2 to reduce mitochondrial membrane potential and increase the release of cytochrome *c*. The levels of cleaved caspase-8 and -9 were significantly increased, which sequentially activated caspase-3 to cleave PARP. We further found that MPEE significantly increased ROS production and activated endoplasmic reticulum (ER) stress associated-apoptotic signaling pathway. Moreover, MPEE significantly inhibited H22 tumor growth in mouse model and improved the survival of tumor mice.

**Conclusion:**

These results suggested that MPEE suppressed hepatocellular carcinoma cell growth through induction of apoptosis via intrinsic- and ER stress-associated pathways.

**Supplementary Information:**

The online version contains supplementary material available at 10.1186/s13020-021-00504-4.

## Introduction

Liver cancer is the sixth most commonly diagnosed cancer and the fourth leading cause of cancer death worldwide in 2018, with about 841,000 new cases and 782,000 deaths annually [1]. About 90% of primary liver cancers are hepatocellular carcinoma (HCC) that causes a major global health problem [2]. The pattern of HCC occurrence shows a large geographical imbalance, with the highest incidence rates in East Asia (more than 50% of the cases occurring in China) [3]. Due to lack of early screening methods, most of patients with HCC were at the advanced stage when they were diagnosed, which led to the poor prognosis. Although a multitude of chemotherapy and targeted therapy agents have been evaluated for the treatment of advanced HCC, such as sorafenib [4, 5], regorafenib [6], and lenvatinib [7], the overall survival benefits are modest. Therefore, the innovative drugs and approaches need to be developed.

Natural products with various structures and biological activities are the treasure of resources for the development of new drugs for cancer treatment. Recently, bryophytes attract lots of interests because they have various biological activities. Many active components including acetogenins, terpenoids and bisbibenzyls have been identified from bryophytes [8] and show different activities, such as antifungal [9], antibacterial [10, 11], antiviral [12], anti-inflammatory [13, 14] and antioxidative [15, 16]. *Marchantia polymorpha* L., a kind of traditional Chinese medicine, distributes worldwide and exhibits antioxidant and antifungal functions [16, 17]. It has been reported that Marchantin A from *M. polymorpha* can inhibit the growth of human MCF-7 breast cancer cells, and increase the levels of cleaved caspase-8, cleaved caspase-3, cleaved caspase-9, and cleaved poly (ADP ribose) polymerase (PARP) [18, 19]. Riccardin D from *M. polymorpha* can be used to treat lung cancer as a DNA topo II inhibitor [20]. However, few research has been done on the anti-hepatoma effect of *M. polymorpha*.

In this study, we prepared *M. polymorpha* ethanol extract (MPEE) and investigated its antitumor effect and mechanism on HCC. We found that MPEE significantly inhibited the growth of HCC cells including BEL-7404, HepG2 and H22 cells through induction of intrinsic- and endoplasmic reticulum (ER) stress-associated apoptosis.

## Materials and methods

### Measurement of flavonoids and polysaccharides in MPEE

*M. polymorpha* was collected from Altay in Xinjiang Uygur Autonomous Region, China. MPEE was prepared according to our previous procedure [21]. Specifically, 100 g powders of *M. polymorpha* were extracted three times using 2 L of 100% ethanol. After centrifugation at 6000 rpm for 15 min, the supernatant was evaporated and freeze-dried using a Freezone 2.5 instrument (Labconco, USA). MPEE was dissolved in DMSO and the contents of flavonoids and polysaccharides were detected according to previous description [22].

### Characterization and quantification of MPEE by LC-QTOF-MS/MS

50 mg of MPEE were applied to extraction procedure, and extracted with 800 μL of methanol included internal standard (2.8 mg/mL, dl-*o*-Chlorophenylalanine). And all samples were grinded to fine powder using Grinding Mill at 65 Hz for 90 s. Then the samples were ultrasonicated for 30 min, by 40 kHz and let stand for 1 h at − 20 °C. The samples were centrifuged at 12,000 rpm and 4 °C for 15 min. 200 μL of supernatant was transferred to vial for LC–MS analysis.

Phytochemical characterization of MPEE was conducted using a quadrupole time-of-flight mass spectrometer (Agilent, 1290 Infinity LC, 6530 UHD and Accurate-Mass Q-TOF/MS), which was coupled with an ultraperformance liquid chromatography system (Waters ACQUITY UPLC, Waters Corp., Milford, MA, USA). Chromatographic separation was achieved using an ODS C18 analytical column (2.5 μm × 210 mm, Waters ACQUITY UPLC@HSS T3). MS conditions were as follows: the scan range was set at *m*/*z* 100–1000. The capillary voltage was 4000 V in positive mode and 3.5 kV in negative mode, the drying gas flow was 11 L/min and the temperature was 350 ℃. The nebulizer pressure was set to 45 psi, the fragmentor voltage was set to 120 V and the skimmer voltage was set to 60 V. The column was kept at 40 ℃, and the flow rate was 0.4 mL/min. The mobile phase solutions consisted of (A) formic acid (0.1%) and (B) acetonitrile: 0.1% formic acid (1:1, v/v). The gradient program was as follows: 0–2 min, 5% B; 2–13 min, 5% B; 13–16 min, 95% B; 16 min, 95% B. All samples were kept at 4 ℃ during the analysis. The injection volume was 4 μL.

The data was performed feature extraction and preprocessed with XCMS in R software, and then normalized and edited into two-dimensional data matrix by Excel 2010 software, including Retention time (RT), Mass-to-charge ratio (MZ), Observations (samples) and peak intensity.

### Animals and ethics statement

Kunming male mice aged 6–8 weeks were housed in a temperature-controlled, light-cycled animal facility of Xinjiang University. These animal studies were authorized by the Committee on the Ethics of Animal Experiments of Xinjiang Key Laboratory of Biological Resources and Genetic Engineering (BRGE-AE001) and carried out in strict accordance with the guide of the Animal Care and Use Committee of College of Life Science and Technology, Xinjiang University. All surgery was performed under sodium pentobarbital anesthesia, and all efforts were made to minimize suffering.

### Cell lines and cell culture

The murine HCC H22 cells, human HCC HepG2 and BEL-7404 cells and the mouse liver NCTC1469 cells were obtained from the Xinjiang Key Laboratory of Biological Resources and Genetic Engineering, Xinjiang University (Urumqi, Xinjiang, China). RPMI 1640 medium (Gibco) was used to culture H22 and BEL-7404 cells, and Dulbecco’s Modified Eagle medium (Gibco) was used to culture HepG2 and NCTC1469 cells. These media were supplemented with 10% heat-inactivated fetal bovine serum (MRC), 1% L-glutamine (100 mM), 100 U/mL penicillin and 100 μg/mL streptomycin. All cells were incubated at 37 °C in a humidified atmosphere of 5% CO_2_.

### MTT assay and cell morphology observation

The inhibitory effects of MPEE on the growth of H22, HepG2, BEL-7404 and NCTC1469 cells were measured by MTT [3-(4,5-dimethyl-2-thiazolyl)-2,5-diphenyl-2-*H*-tetrazolium bromide] (Sigma, MO, USA) assay according to our previous description [21]. Briefly, H22, HepG2, BEL-7404 and NCTC1469 cells at the density of 5 × 10^4^ cells/mL were seeded in 96-well plates and cultured overnight. Cells were treated with different concentrations (0, 25, 50, 70, 100 or 200 μg/mL) of MPEE for 24 h or 48 h. DMSO (0.6%) and cisplatin (30 μg/mL) were used as negative or positive controls, respectively. Six wells were repeated for each treatment. Splenocytes (1 × 10^6^ cells/mL) from C57BL/6 mice were seeded in 96-well plates and treated with different concentrations of MPEE for 24 h and 48 h. The relative cell viability was determined as: Cell viability (%) = (OD_treated_/OD_untreated_) × 100%.

After treatment with MPEE for 24 h and 48 h, the morphology of H22 cells was observed by inverted fluorescence microscope (Nikon Eclipse Ti-E, Japan).

### Detection of cell cycle

H22 cells were inoculated in 60 mm culture dishes at the density of 5 × 10^4^ cells/mL and treated with different concentrations of MPEE for 24 h. Cells were harvested and fixed with 70% ethanol at 4 °C overnight. After washing with cold PBS, cells were stained with propidium iodide (PI) as described [23]. Samples were analyzed by flow cytometry (BD FACSCalibur, CA, USA) and the cell cycle distribution was analyzed using ModFit LT 3.0 software.

### Analysis of cell apoptosis

H22, BEL-7404 and HepG2 cells were treated with different concentrations of MPEE for 24 h and stained with apoptosis detection kit (YEASEN, China) according to the manufacturer’s instructions. DMSO and cisplatin were used as negative and positive controls, respectively. For the inhibitor experiment, H22 cells were pretreated with 15 μM Z-VAD-FMK and 20 μM Ac-DEVD-CHO (Beyotime, China) for 2 h, then treated with MPEE for 24 h. Samples were analyzed by flow cytometry.

### Hoechst 33258, JC-1 and DCFH-DA staining

H22 cells were seeded in 6-well plate at the density of 5 × 10^4^ cells/mL. After 60% ~ 70% confluence, the cells were treated with MPEE for 24 h. The cells were collected and fixed with 4% ice-cold Paraformaldehyde at 4 °C for 10 min. After washing with PBS, H22 cells were stained with Hoechst 33258, JC-1 dye or 2,7 dichlorodihydrofluoresc-ein diacetate (DCFH-DA) (Beyotime, China) as previously described [23]. Samples were observed by an inverted fluorescence microscopy (Nikon, Japan) or analyzed by flow cytometry.

### Migration in vitro

The migration of H22 cells in *vitro* was tested by wound healing assay as described [24]. H22 cells (2.5 × 10^4^/well) were seeded in a 24-well plate. A vertical wound with uniform size was scratched through the center of each well using a 200 μL pipette tip. After treatment with MPEE for 24 h and 48 h, the average distances of cell migration were analyzed by Image J.

### Western blot

The antibodies against caspase-9, Bax, Bcl-2, PERK, eIF2α and ATF6, the phosphorylation antibodies of PERK and eIF2α, anti-mouse IgG-HRP and anti-rabbit IgG-HRP were purchased from BBI Life Sciences (Shanghai, China). The antibodies against caspase-3, caspase-8, PARP, cytochrome *c* and β-actin were obtained from Cell Signaling Technology (Danvers, MA, USA). The antibodies against CHOP, cyclinB1, cdk2 and cyclin D1were bought from Beyotime (Shanghai, China).

After treatment with MPEE for 24 h, total protein of H22 cells was isolated by RIPA Lysis Buffer (Beijing ComWin Biotech Co., Ltd) and the protein concentration was detected by the bicinchoninic acid assay kit (Thermo Fisher Scientific, USA) according to the manufacturer’s instructions. Equal amount of proteins were separated on 12% SDS-PAGE and then transferred onto PVDF membrane. The membrane was blocked with TBST buffer (20 mmol/L Tris–HCl, 150 mmol/L NaCl, 0.05% Tween 20) contained 5% skim milk for 1 h at room temperature, and incubated with primary antibodies overnight at 4 °C on a gentle shaker. After washing with TBST buffer three times, the membrane was incubated with secondary antibodies for 2 h. The target proteins were visualized using a commercial ECL kit (Beyotime).

### Quantitative RT-PCR (qRT-PCR)

H22 cells were treated with MPEE for 24 h and the total RNA was extracted by TRIzol reagent (Invitrogen, Carlsbad, CA, USA) according to the manufacturer’s protocol. Reverse transcription and quantitative PCR were carried out using reverse transcriptase M-MLV (Takara, China) and TransStart Tip Green qPCR SuperMix Kit (TransGen Biotech, China), respectively. The gene-specific primers were shown in Table 1.

**Table 1:** The gene-specific primers

Gene	Primer sequences (5′–3′)	
*GAPDH*	F: AGCCTCGTCCCGTAGACA	R: CTCGCTCCTGGAAGATGG
*Srp72*	F: GAGGGGTCGACATTGCTCTC	R: GCCAGTTAAAGACCTCCCCC
*Srp14*	F: GCAAACCAGCACAGTGACAG	R: ACAACTAGCCCAAGCCCATC
*Srprb*	F: TCAGCTCCTGTTGTGTCACC	R: ATGCAGCGATCTGTAGGCTC
*Srpr*	F: AGAGCCTTGGCTGACCATTC	R: GCCAGTACCCACAAAGACGA
*Srp68*	F: CCAAACAAGCCAACCTCGTG	R: TGCCCTTGATGTAGCCTGTG
*Srp19*	F: TGCTCAGCAGTTGGACTGAAT	R: TTGCTGAAGACTTGGGTCGG
*Wfs1*	F: GGAAACTAACATGGCCCGGA	R: TCCAATCGCCCAGGAAGAAC
*Atf6*	F: AAGGGTCAACCAGGGATACG	R: AAACACCCACAAGCCACAGG
*Gadd34*	F: GAGAAGACCAAGGGACGTGG	R: TCGATCTCGTGCAAACTGCT
*Hspa5*	F: GTGTGTGAGACCAGAACCGT	R: TAGGTGGTCCCCAAGTCGAT
*Rpl22l1*	F: ATGGCGCCGCAGAAAGACA	R: GACCACACGTAGCCAATCACG
*Rps29*	F: AGCCGACTCGTTCCTTTCTC	R: TTCAGCCCGTATTTGCGGAT
*Rpl13a*	F: CGGCTGAAGCCTACCAGAAA	R: GGAGTCCGTTGGTCTTGAGG
*Cyclin B1*	F: AAGGCCAAGGTCAGTATGGC	R: CTCAGGCTCAGCAAGTTCCA
*Cyclin D1*	F: AGGCAGCGCGCGTCAGCAGCC	R: TCCATGGCGCGGCCGTCTGGG
*Cdk2*	F: CACAGGGCTTGCACGTCACT	R: TGTCTCCTGGCCTGCATCAC
*Ddit3*	F: GCAGCGACAGAGCCAGAATA	R: ATGTGCGTGTGACCTCTGTT
*Cdc25b*	F: ATCCTTACCAGTGAGGCTGC	R: CTCTGGAAGCGCACATTCTC
*Mcm4*	F: CAAGGTTCAACCAGGGGACA	R: ATGCAGACGTTTTGCATCCG
*Mcm2*	F: AGAAGTTCAGCGTCATGCGGAGTA	R: CCCAAAGCGGTTGCGTTGATATGT
*Cdk1*	F: AAGTGTGGCCAGAAGTCGAG	R: AAAGTACGGGTGCTTCAGGG
*Gadd45α*	F: CTGCAGAGCAGAAGACCGAA	R: GCAGGCACAGTACCACGTTA
*Bax*	F: GCCTCCTCTCCTACTTC	R: CCTCAGCCCATCTTCTT
*Bcl-2*	F: CACTTGCCACTGTAGAGA	R: GCTTCACTGCCTCCTT

### Tumor mouse study

Kunming male mice were subcutaneously injected with H22 cells (1 × 10^6^ cells/mice) into the right flank and randomly divided into 5 groups (8 mice/group). After 6 days, tumor mice were intraperitoneally treated with MPEE (50 mg/kg or 100 mg/kg in 0.1 mL DMSO) every two days for 10 times. Cisplatin (5 mg/kg) was intraperitoneally injected every three days for 7 times and 0.1 mL PBS was intraperitoneally injected every two days for 10 times, which were used as positive and negative controls, respectively. Tumor sizes were measured using calipers and calculated according to the following formula: tumor volume (mm^3^) = (length × width^2^)/2. On day 57, the survival rates of tumor mice in each group were calculated with Prism 5.

### Statistical analysis

Statistical significance was calculated by one-way analysis of variance. All data were expressed as the mean ± standard error of the mean (SEM). *p* < 0.05 was considered statistically significant.

## Results

### MPEE reduced the viability of HCC cells

**Fig. 1:**
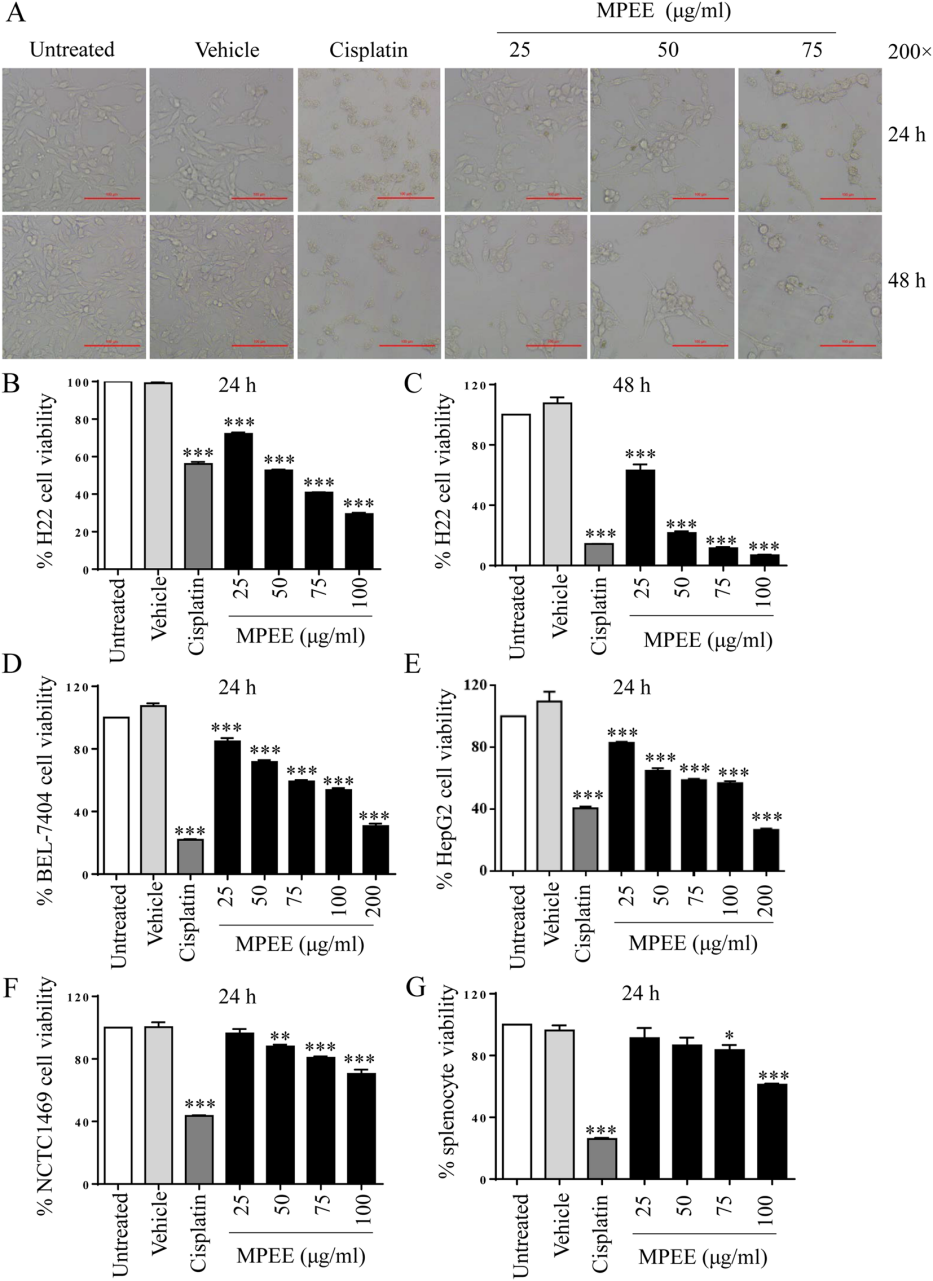
Effects of MPEE on the proliferation of H22, BEL-7404, HepG2 and NCTC1469 cells and splenocytes. **A** After MPEE treatment for 24 h and 48 h, the morphological changes of H22 cells were observed by inverted microscope. **B**–**C** The viability of H22 cells was measured by MTT assay after MPEE treatment for 24 and 48 h. **D**–**F** The viability of BEL-7404, HepG2 and NCTC1469 cells after MPEE treatment for 24 h. **G** The viability of splenocytes from C57BL/6 mice after MPEE treatment for 24 h. Data were analyzed by ANOVA. *p < 0.05; **p < 0.01; ***p < 0.001 compared to untreated group

MPEE contained 42.5% of polysaccharides and 5.6% of flavonoids. The inhibitory effect of MPEE on the proliferation of HCC cells was determined by inverted microscope and MTT assay. After treatment with different concentrations (0, 25, 50, 75 and 100 μg/mL) of MPEE and cisplatin for 24 and 48 h, H22 cells showed small and round morphology, and cell numbers were enormously decreased (Fig. 1A). Compared to untreated cells, the viability of H22 cells was dose- and time-dependently decreased and the IC50 values were 53.5 μg/mL at 24 h and 30.8 μg/mL at 48 h (Fig. 1B, C). Moreover, the viability of BEL-7404 and HepG2 cells was also dose-dependently reduced by MPEE treatment and the IC50 values for BEL-7404 and HepG2 cells were 108.4 μg/mL and 118.4 μg/mL at 24 h, respectively (Fig. 1D, E). Although MPEE reduced the viability of normal liver NCTC1469 cells, the IC50 value (168.9 μg/mL) is much higher than that of HCC cells (Fig. 1F). Moreover, the effect of MPEE on the viability of murine splenocytes was also detected. We found that MPEE had low cytotoxic effect on splenocytes (Fig. 1G). The results suggested that MPEE significantly reduced the viability of HCC cells with low cytotoxicity on normal cells.

### MPEE induced cell cycle arrest in H22 cells

**Fig. 2:**
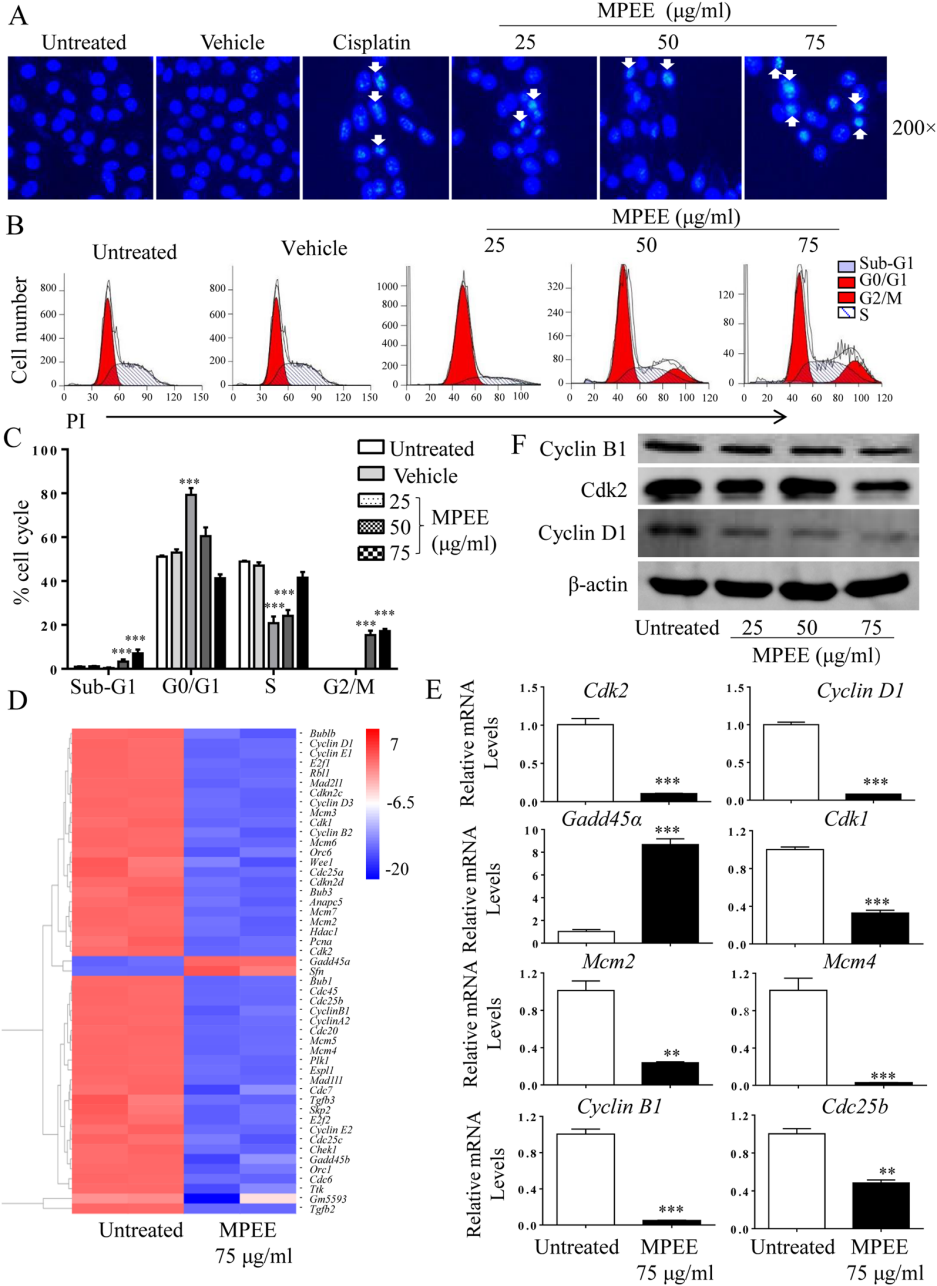
Nuclear morphology and cell cycle distribution of H22 cells upon MPEE treatment. H22 cells were treated with different concentrations of MPEE for 24 h. **A** After staining with Hoechst 33258, nuclear morphology of H22 cells was observed by inverted fluorescence microscopy. The arrows indicated the chromosomal condensation. **B**–**C** Cell cycle phase distribution was analyzed by flow cytometry following PI staining. **D** Heatmap of clustered cell cycle associated genes as evaluated by transcriptome analysis. **E** The mRNA levels of *Cdk2*, *Cyclin D1*, *Gadd45α*, *Cdk1*, *Mcm2*, *Mcm4*, *Cyclin B1* and *Cdc25b* were analyzed by qRT-PCR. **F** The protein levels of Cyclin B1, Cyclin D1 and Cdk2 were detected by Western blot. Data were analyzed by ANOVA. **p < 0.01; ***p < 0.001 compared to untreated group

The nuclear morphology of H22 cells was further detected using Hoechst 33258 staining after treatment with MPEE for 24 h. The nuclei of MPEE-treated cells showed chromatin condensation and fragmentation, while the nuclei of untreated cells showed homogeneous staining (Fig. 2A). To investigate whether MPEE induces cell cycle arrest in H22 cells, cells were treated with different concentrations (0, 25, 50 and 75 μg/mL) of MPEE for 24 h and stained with PI. As shown in Fig. 2B, C, cell cycle was arrested at G0/G1 phase upon low concentration of MPEE treatment, while it was arrested at G2/M phase upon high concentrations of MPEE treatment. The proportions of sub-G1 cells were also significantly increased in a dose-dependent manner.

The expression of cell cycle-related genes was analyzed by transcriptome analysis and verified by qRT-PCR. After treatment with 75 μg/mL MPEE for 24 h, total RNA was isolated to carry out transcriptome analysis. Most genes related to cell cycle were down-regulated except Gadd45α and Sfn (Fig. 2D). qRT-PCR was used to verify the expression of Cdk2, Cyclin D1, Cdk1, Mcm2, Mcm4, Cyclin B1, Cdc25b and Gadd45α, which was consistent with transcriptome analysis (Fig. 2E). The protein levels of Cyclin B1, Cdk2 and Cyclin D1 were also significantly reduced by MPEE treatment in a dose-dependent manner (Fig. 2F; Additional file 1: Fig. S1). The results showed that MPEE induced cell cycle arrest through regulating the expression of cell cycle-related genes.

### MPEE induced apoptosis of HCC cells

**Fig. 3:**
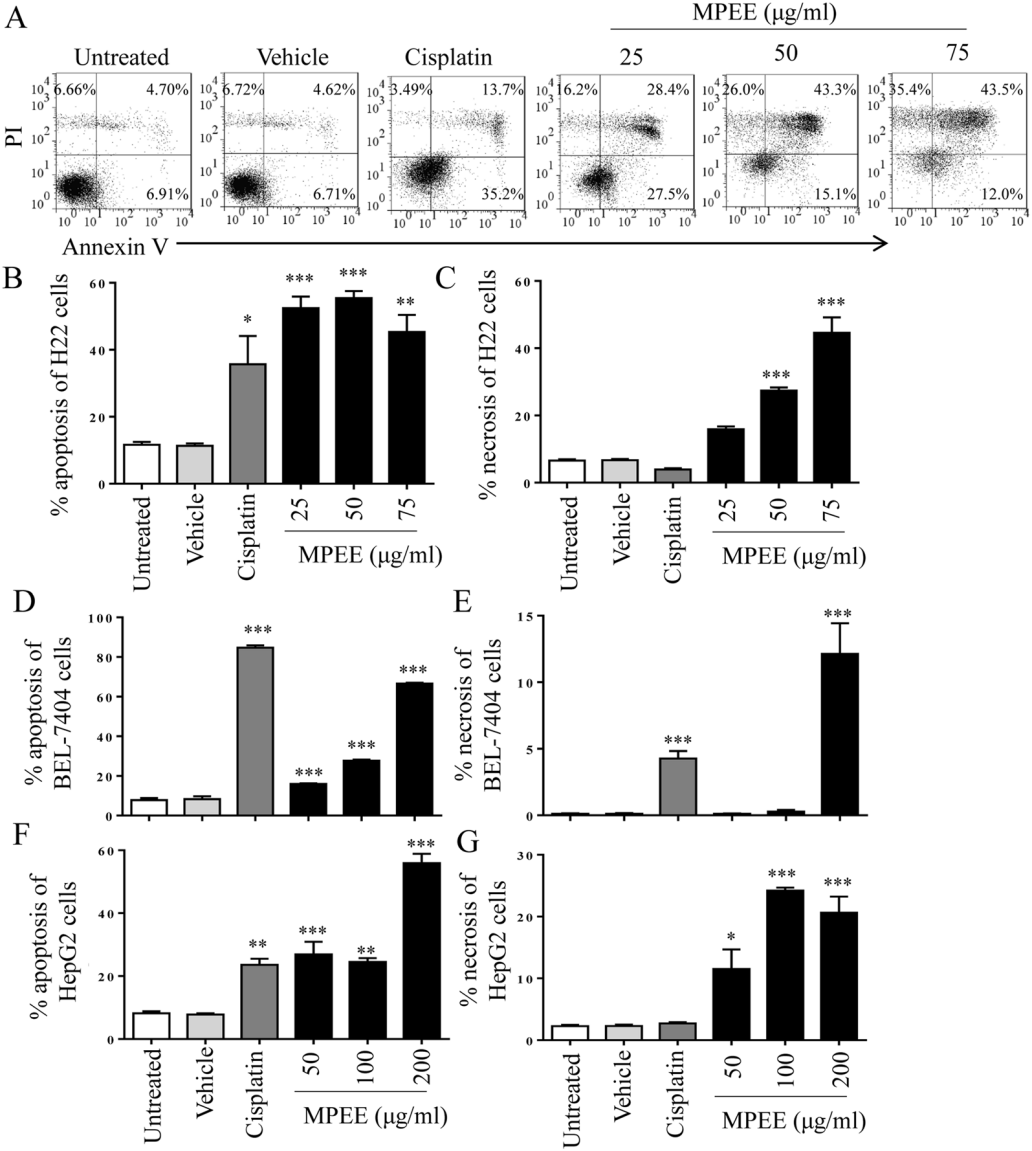
The apoptosis of H22, BEL-7404 and HepG2 cells induced by MPEE treatment. Different concentrations of MPEE were used to treat H22, BEL-7404 and HepG2 cells for 24 h. **A**–**C** The apoptosis and necrosis of H22 cells were analyzed by flow cytometry following Annexin V/PI staining. **D**–**G** The apoptosis and necrosis of BEL-7404 and HepG2 cells were shown. Data were analyzed by ANOVA. *p < 0.05; **p < 0.01 ***p < 0.001 compared to untreated group

MPEE caused the chromatin condensation and fragmentation that was the characterization of apoptosis. Therefore, the apoptosis of HCC cells was analyzed by Annexin V-FITC and PI staining after treatment with different concentrations (0, 25, 50 and 75 μg/mL) of MPEE for 24 h. The results showed that the percentages of apoptosis H22 cells including early (AnnexinV^+^PI^−^) and late (AnnexinV^+^PI^+^) apoptosis were significantly increased after MPEE treatment (Fig. 3A, B). The similar results were observed in BEL-7404 and HepG2 cells (Fig. 3D, F). Although MPEE also induced necrosis (AnnexinV^−^PI^+^) in HCC cells (Fig. 3C, E, G), it mainly induced apoptosis. The results indicated that MPEE induced apoptosis of HCC cells.

### MPEE activated mitochondria-dependent apoptosis pathway

**Fig. 4:**
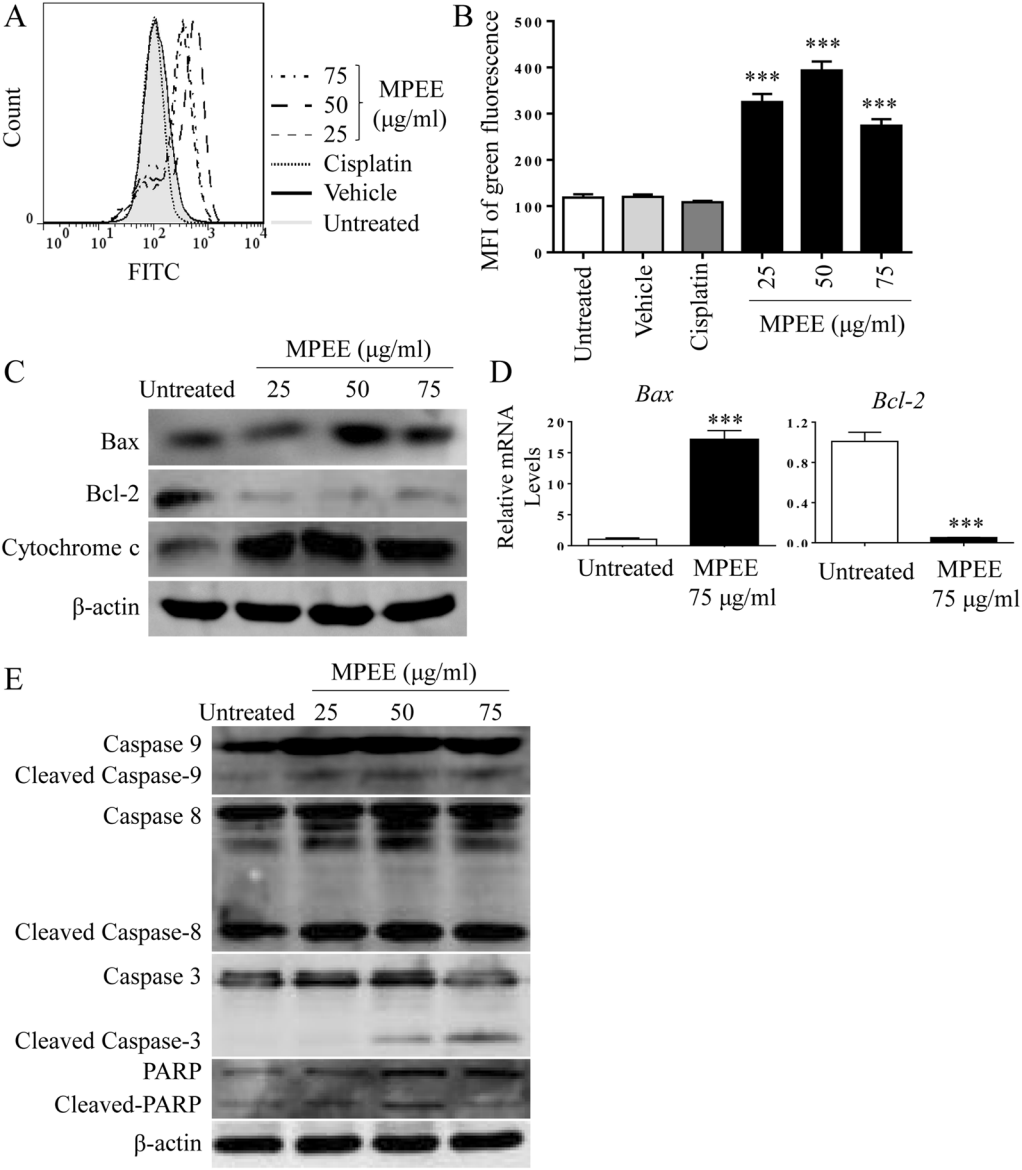
The effects of MPEE on Δψ_m_ and caspase cascade in H22 cells. H22 cells were treated with different concentrations of MPEE for 24 h. **A**, **B** Cells were stained with JC-1 and the fluorescence changes were analyzed by flow cytometry. **C** The protein levels of Bax, Bcl-2 and cytochrome *c* were detected by Western blot. **D** The mRNA levels of *Bax* and *Bcl-2* were analyzed by qRT-PCR. **E** The levels of cleaved-caspases and -PARP were detected by Western blot. Data were analyzed by ANOVA. ***p < 0.001 compared to untreated group

Mitochondrial membrane potential (Δψm) plays a critical role in the activation of intrinsic apoptosis pathway, which can be monitored by JC-1 staining. Red and green fluorescences represent JC-1 aggregate and monomer, respectively and the increase of green fluorescence indicates the reduction of Δψm. H22 cells were treated with MPEE for 24 h and stained with JC-1 dye. We found that the green fluorescence in H22 cells was significantly enhanced by MPEE treatment (Fig. 4A, B), indicating that Δψm was lessened. Δψm is strictly regulated by proteins of the B cell lymphoma 2 (Bcl-2) family including anti-apoptotic Bcl-2 and pro-apoptotic Bcl-2-associated X protein (Bax). Therefore, the RNA and protein levels of Bax and Bcl-2 were detected by qRT-PCR and Western blot after MPEE treatment for 24 h. Consistently, the levels of Bax and Bcl-2 were increased and decreased at both mRNA and protein levels, respectively (Fig. 4C, D; Additional file 1: Fig. S1), which caused the reduction of Δψm. Depletion of Δψm leads to the release of cytochrome *c* into the cytoplasm to initiate apoptosis cascade [25]. After treatment with MPEE for 24 h, total protein of H22 cells was isolated to test the levels of cytochrome *c* by Western blot. Consistently, the levels of cytochrome *c* were greatly increased upon MPEE treatment (Fig. 4C; Additional file 1: Fig. S1). We subsequently measured the activation of caspase cascade induced by mitochondria-dependent pathway and found that the levels of cleaved caspase-9 and -3 were greatly increased by MPEE treatment compared with the untreated control. At the same time, MPEE promoted the cleavage of caspase-8 (Fig. 4E; Additional file 1: Fig. S1). Sequentially, the upregulated level of cleaved DNA repair enzyme of poly (ADP-ribose) polymerase (PARP) was observed. The results suggested that caspase cascade was involved in the apoptosis induced by MPEE.

**Fig. 5:**
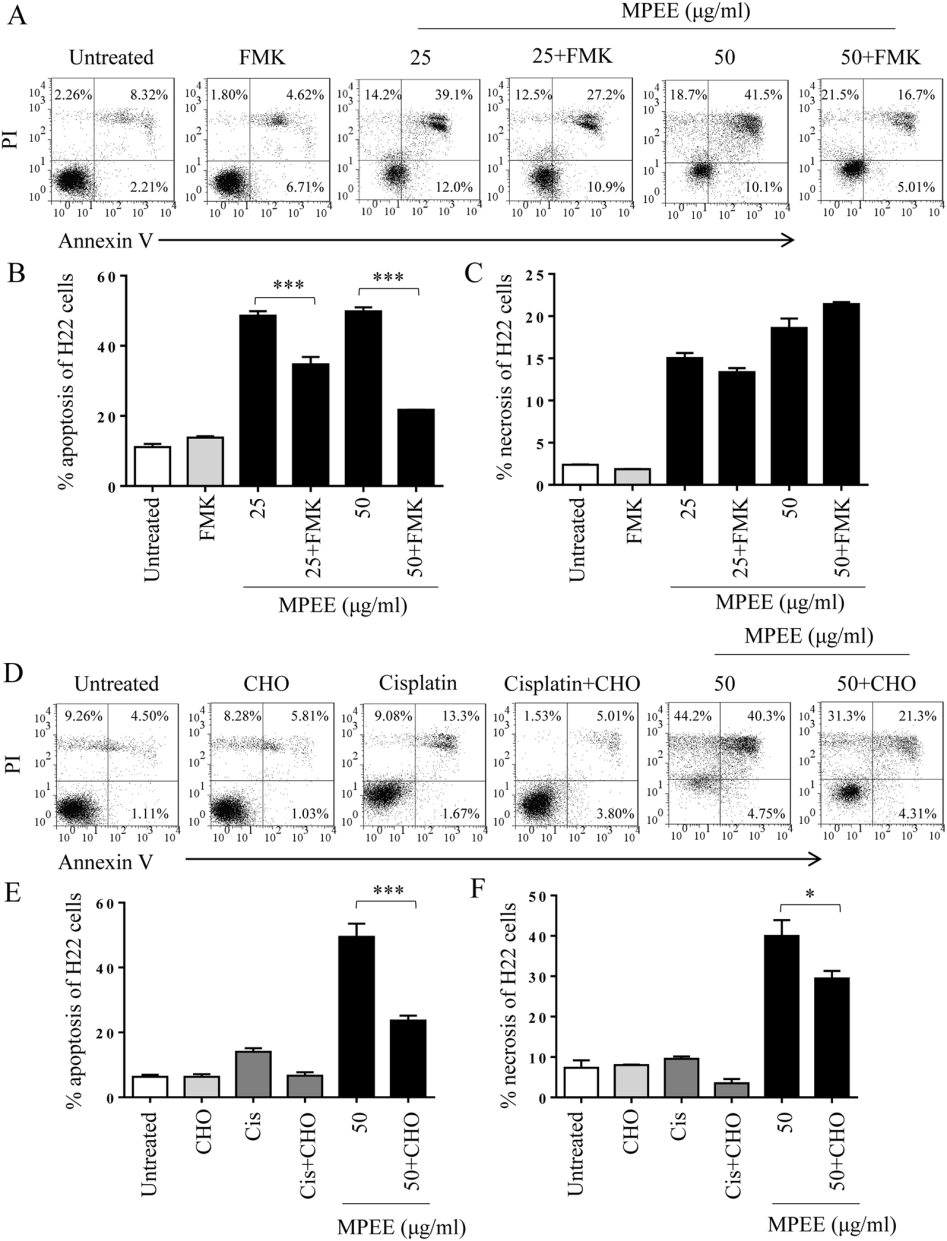
The effect of caspase inhibitors on apoptosis of H22 cells induced by MPEE. H22 cells were pretreated with 15 μM FMK or 20 μM CHO for 2 h, and then treated with MPEE. After 24 h, apoptosis and necrosis of H22 cells were analyzed by flow cytometry. FMK pretreatment was shown in **A**–**C** and CHO pretreatment was shown in **D**–**F**. Data were analyzed by ANOVA. *p < 0.05; ***p < 0.001 compared to untreated group

To investigate the role of caspase in the induction of apoptosis, H22 cells were pretreated with Z-VAD-FMK (FMK, a broad-spectrum caspase inhibitor) and Ac-DEVD-CHO (CHO, a caspase 3 inhibitor), and then treated with MPEE. After 24 h, the apoptosis of H22 cells was analyzed by flow cytometry. The pretreatment of FMK and CHO significantly decreased the apoptosis of H22 cells induced by MPEE (Fig. 5A–F), suggesting that mitochondria-dependent pathway partially mediated MPEE-induced apoptosis.

### MPEE induced reactive oxygen species (ROS) production and endoplasmic reticulum (ER) stress

**Fig. 6:**
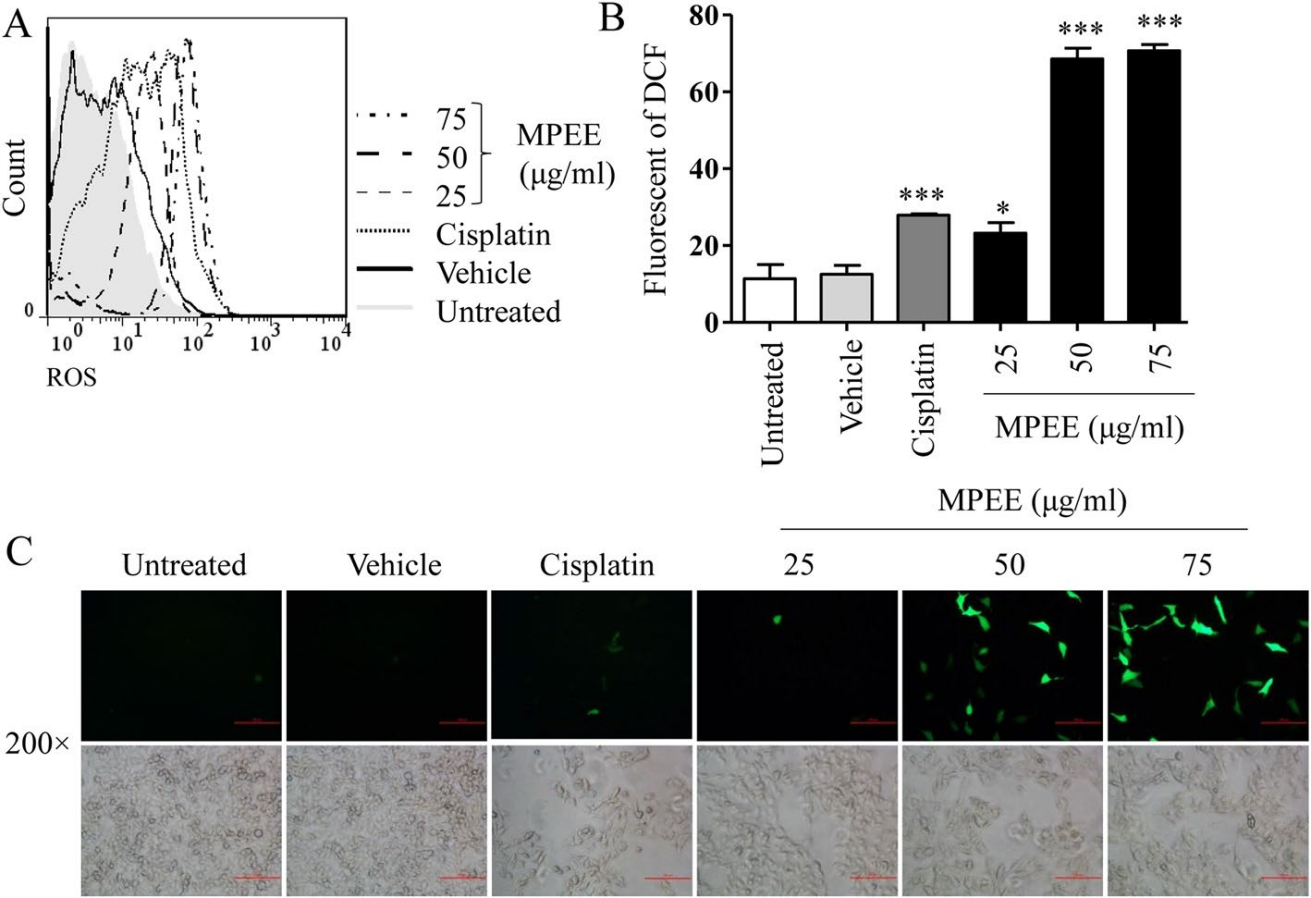
ROS production in H22 cells induced by MPEE. H22 cells were treated with different concentrations of MPEE for 24 h and stained with DCFH-DA. **A**, **B** Samples were analyzed by flow cytometry. **C** Samples were observed using inverted fluorescence microscopy. Data were analyzed by ANOVA. *p < 0.05; ***p < 0.001 compared to untreated group

**Fig. 7:**
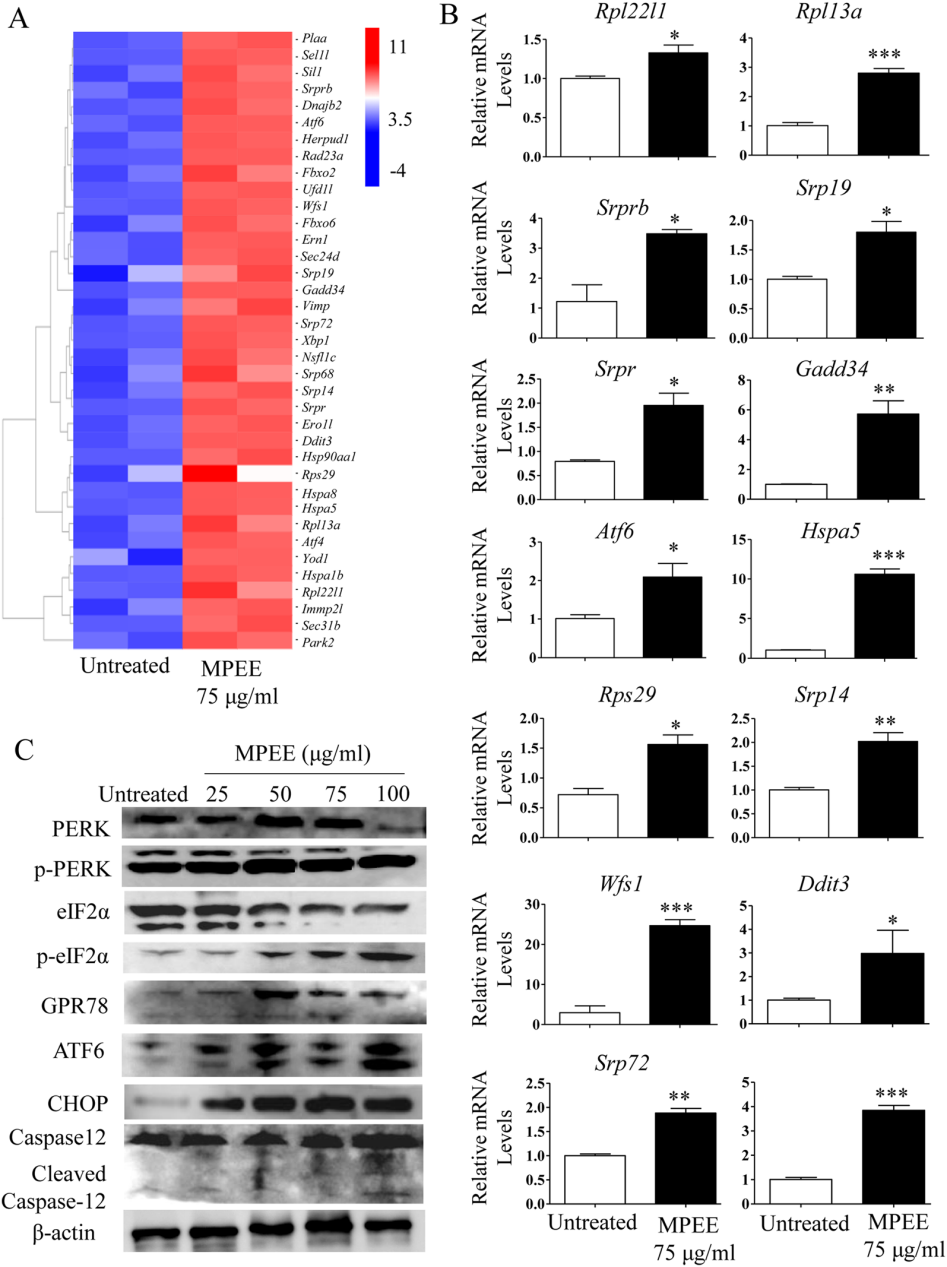
MPEE activated ER stress in H22 cells. H22 cells were treated with MPEE for 24 h and the total RNA was isolated. **A** Heatmap of clustered ER stress-associated genes as evaluated by transcriptome analysis. **B** The mRNA levels for *Rpl22l1*, *Rpl13a*, *Srprb*, *Srp19*, *Srpr*, *Gadd34*, *Atf6*, *Hspa5*, *Rps29*, *Srp14*, *Wfs1*, *Ddit3*, *Srp72* and *Srp68* were analyzed by qRT-PCR*.* **C** The levels of ER stress-associated proteins were analyzed by Western blot. Data were analyzed by ANOVA. *p < 0.05; **p < 0.01; ***p < 0.001 compared to untreated group

It has been reported that ROS production was involved in the induction of mitochondrial dysfunction and ER stress [26]. We found that MPEE significantly induced ROS production using both flow cytometry and inverted fluorescence microscopy after treatment for 24 h (Fig. 6A–C). Consistently, the transcriptome analysis showed that MPEE significantly up-regulated 70 genes related to protein processing in ER and 53 genes related to Ribosome (Fig. 7A), suggesting that ER stress signaling pathway was activated. The expression of Rpl22l1, Rpl13a, Rps29, Srp14, Srprb, Srp19, Srp72, Srp68, Srpr, Gadd34, Wfs1, Ddit3, Atf6 and Hspa5 was verified by qRT-PCR, which was consistent with transcriptome analysis (Fig. 7B).

We further investigated whether the ER stress pathway was involved in apoptosis induced by MPEE in H22 cells. After treatment with different concentrations of MPEE for 24 h, the level of phosphorylated protein kinase-like ER kinase (p-PERK) was significantly increased (Fig. 7C; Additional file 2: Fig. S2). PERK releases glucose-regulated protein 78 (GRP78/BiP) and phosphorylates eukaryotic translation initiation factor 2 alpha (eIF2α), which lead to a general decrease in protein translation [27]. We found that the phosphorylation of eIF2α and the level of GPR78 were up-regulated by MPEE treatment. In addition, activating transcription factor 6 (ATF6), an ER type II transmembrane protein, was also up-regulated. ATF6 entered the nucleus to activate the expression of GRP78 and C/EBP homologous protein (CHOP) genes. We also found that MPEE significantly increased the levels of CHOP (Fig. 7C; Additional file 2: Fig. S2). The results indicated that MPEE might induce apoptosis in H22 cells through ER stress signaling pathway.

### MPEE suppressed in vitro migration and in vivo growth of H22 cells

**Fig. 8:**
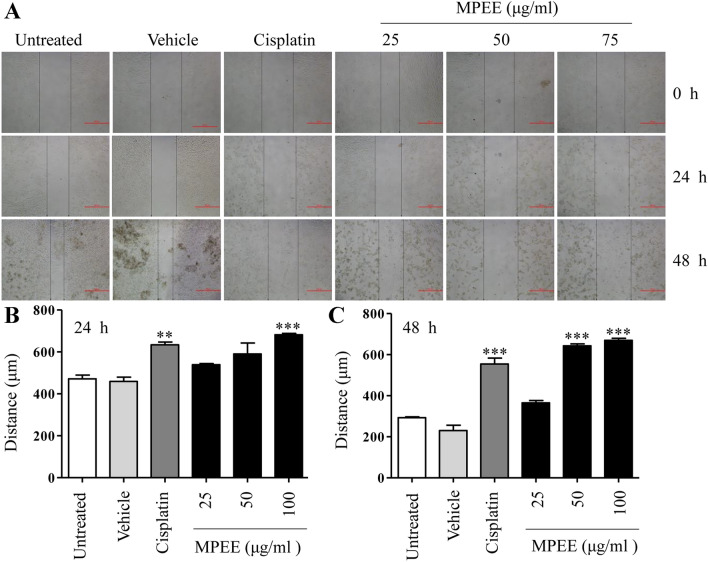
MPEE suppressed the migration of H22 cells in vitro. H22 cells were treated with different concentrations of MPEE for 24 h and 48 h. The migration of H22 cells was observed by inverted microscope (**A**) and analyzed by Image J (**B**, **C**). Data were analyzed by ANOVA. **p < 0.01; ***p < 0.001 compared to untreated group

**Fig. 9:**
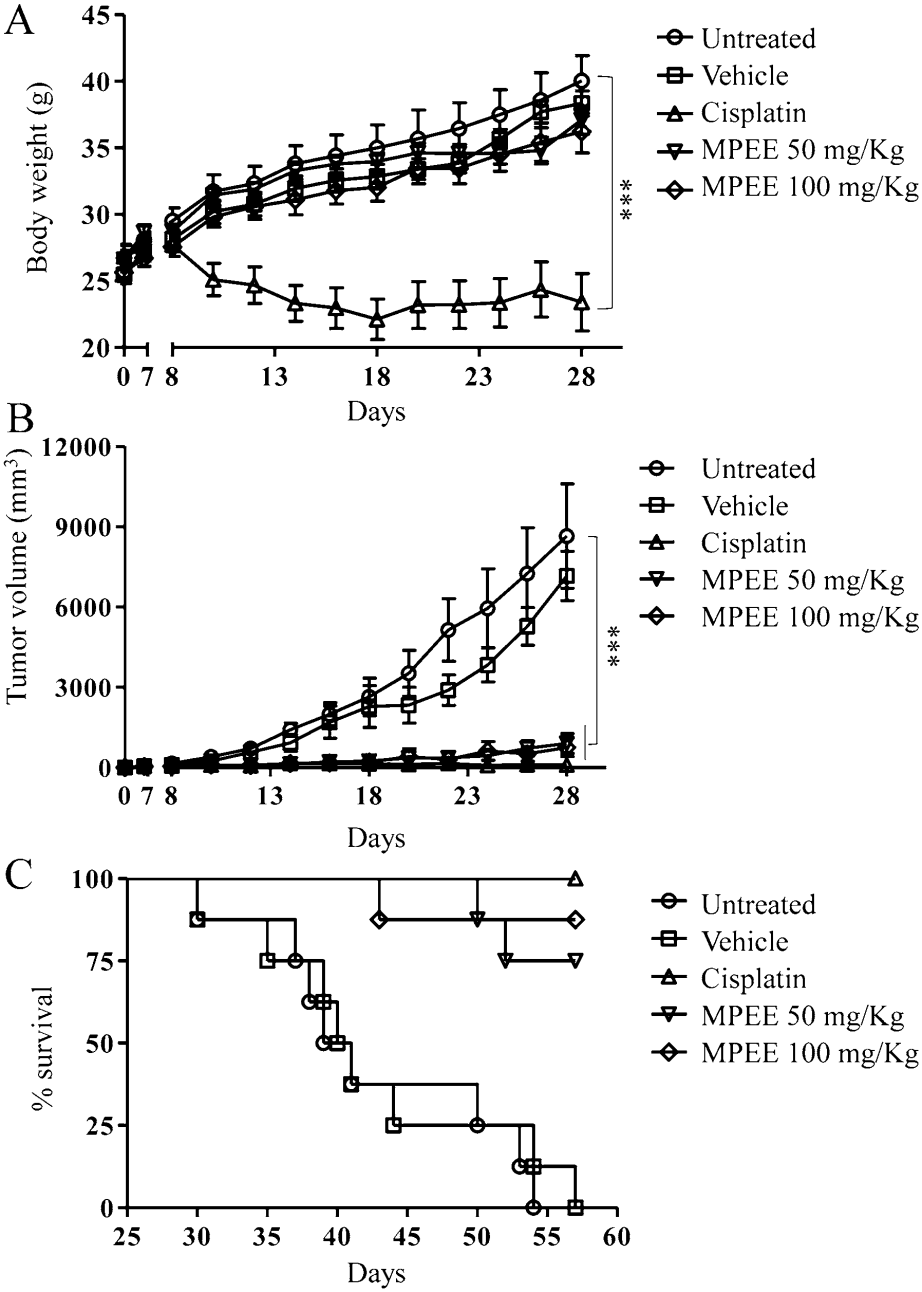
MPEE inhibited H22 tumor growth in vivo. Tumor mouse model was established by injection of H22 cells. After 6 days, tumor mice (8 mice/group) were intraperitoneally treated with DMSO, cisplatin and MPEE. Body weight and tumor volumes were shown in **A** and **B**, respectively. **C** The survival rate of tumor mice was monitored. Data were analyzed by ANOVA. ***p < 0.001 compared to model group

Wound healing method was used to determine the migration of H22 cells in vitro. We found that MPEE significantly suppressed H22 cell migration in a dose-dependent manner (Fig. 8A–C). H22 tumor mouse model was further used to evaluate the antitumor effect of MPEE. After 6 days of H22 cell injection, tumor mice were intraperitoneally treated with DMSO, cisplatin and MPEE. The body weight of mice and tumor sizes were monitored at indicated time points. Compared with untreated and DMSO groups, cisplatin significantly reduced the body weight but MPEE did not significantly change the body weight, suggesting that the selected doses of MPEE had no obvious side effect (Fig. 9A). Interestingly, the tumor growth in mice treated with both 50 and 100 mg/kg of MPEE was significantly inhibited (Fig. 9B). Moreover, both doses of MPEE greatly improved the survival of tumor mice (50 mg/kg: 6 out of 8; 100 mg/kg: 7 out of 8) compared with model groups (0 out of 8) at the end of the experiment (Fig. 9C). The results showed that MPEE suppressed H22 cell growth in vivo and improved the survival of tumor mice.

### Qualitative and quantitative analysis of the active ingredients in MPEE

The MPEE was characterized by LC-Q-TOF–MS and compounds were identified according to mass spectrometry data under both negative and positive ESI mode (Additional file 3: Fig. S3). 67 ingredients with the relative content more than 100 ng were found under negative ESI mode, which included nine fatty Acyls, eight flavonoids and four benzopyrans [28–48] (Additional file 4: Table S1). The most abundant component is 3,5,7-trihydroxy-2-(3-hydroxyphenyl)-4*H*-chromen-4-one, which belongs to flavonoids with molecular weight of 286.04 and retention time of 6.74 min. Meanwhile, compound identification was performed according to mass spectrometry data under positive ESI mode (Additional file 3: Fig. S3), 20 ingredients with the relative content more than 50 ng were identified under positive ESI mode (Additional file 5: Table S2), which included two flavonoids, one isoflavonoids, two prenol lipids, one kind of steroids and steroid derivatives, coumarins and derivatives and stilbenes [49–54]. The most abundant component is beta-patchoulene, which belongs to polycyclic hydrocarbons with molecular weight of 204.19 and retention time of 12.06 min.

## Discussion

Compared with conventional chemotherapeutics, natural compounds can exert potent antitumor effect with or without minor adverse effects [55]. A number of plant-derived natural products have been investigated for their antitumor activities [21, 23, 56]. Recently, it has been reported that bryophytes can induce apoptosis and cell cycle arrests [19, 57]. In this study, our results showed that MPEE inhibited HCC cell growth both in vitro and in vivo, which might induce cell cycle arrest and apoptosis of HCC cells through intrinsic- and ER stress-associated signaling pathways.

The antiproliferative activity of MPEE was first examined. The results showed that MPEE significantly inhibited the growth of H22, HepG2 and BEL-7404 cells. Cellular proliferation is primarily controlled by the cell cycle, which consists of four sequential phases (G0/G1, S, G2, and M) [58]. Cyclin-dependent kinases (CDKs) and the cyclins are the key regulators of cell cycle transition [59, 60]. Cdk2 regulates the cell cycle transition from G1 to S phase [61]. Cyclin D1 is another regulator that drives G1 to S phase progression and its dysregulation can be frequently found in human cancers including HCC [62]. Cyclin B is mainly involved in the completion of M phase [63]. In our study, we observed that low concentrations of MPEE treatment significantly induced H22 cell cycle arrest at G0/G1phase, and decreased the expression of Cdk2 and cyclin D1 at both levels of mRNA and protein. However, high concentrations of MPEE arrested H22 cells at G2/M phase with a significant decrease of cyclin B expression, which might be due to the different components of MPEE to induce the cell cycle arrest at the different phases. Consistently, MPEE significantly downregulated the expression of Cdk1, which plays an important role in the transition from G2 to M phase [64]. It has been reported that Cdc25b activates Cdk1/cyclinB but growth arrest and DNA damage-inducible 45 alpha (Gadd45a) inhibits the activation of Cdk1and Cdk1-cyclinB complex [65]. We also found that MPEE downregulated and upregulated the expression of Cdc25b and Gadd45a, respectively. The results indicated that MPEE suppressed the growth of HCC cells by the induction of cell cycle arrest.

Minichromosome Maintenance (MCM) family is essential for DNA replication in each cell cycle. Mcm4 affects the DNA helicase activity of the Mcm2–7 complex. Mcm2 is associated with the progression from cirrhosis to HCC and poor cellular differentiation. MCMs were significantly up-regulated in HCC [66]. We observed that MPEE significantly reduced the expression of Mcm2 and Mcm4, suggesting that MPEE might suppress the growth of HCC cells through interference of DNA replication. It has been reported that cyclin D1 not only regulates the transition from G1 to S phase but also promotes tumor invasion and metastasis, and cyclin D1 deletion can reduce the migration of tumor cells [67]. Similarly, MPEE inhibited H22 cell migration in vitro, suggesting that MPEE might inhibit tumor invasion and metastasis.

Apoptosis also plays a crucial role for controlling the proliferation of cancer cells and has been considered as a major route to eradicate cancer cells [68]. Both caspase-independent and -dependent pathways can account for the programmed cell death [69, 70]. Caspase-dependent apoptosis can be induced by the intrinsic (mitochondria-dependent) pathway and the extrinsic (death receptor) pathway [71]. The loss of Δψm is the major characteristic of mitochondria-dependent apoptosis because it promotes the release of cytochrome *c* from mitochondria to cytosol and activation of caspase-9. We found that MPEE reduced Δψm of HCC cells and increased the release of cytochrome *c*, which activated caspase-9. At the same time, MPEE also activated caspase-8. Therefore, both active caspase-9 and -8 might activate caspase-3 to degrade PARP. We further observed that both broad-spectrum caspase inhibitor and caspase 3 inhibitor significantly reduced apoptosis induced by MPEE. The results indicated that MPEE induced apoptosis in HCC cells through both intrinsic signaling pathways.

ER is well known to regulate cellular responses to stress. Aberrant accumulation of misfolded/unfolded proteins, oxidative stress and Ca^2+^ imbalance can activate ER stress [72, 73], which is involved in the induction of apoptosis [74]. ER stress-associated apoptosis in cancer cells represents the potential target for the development of cancer therapeutic drugs. We found that MPEE dramatically increased the ROS production in HCC cells, which might contribute to the activation of ER stress. The transcriptome analysis showed that a large number of up-regulated genes including Atf6, Gadd34, Rps29, Srp14, Srp19, Srp72, and Srp68 were enriched in ribosome, protein export and ER stress-related signaling pathways [75]. These data suggested that MPEE induced ER stress in HCC cells. ER stress can activate the unfolded protein response (UPR), which includes PERK, ATF6 and inositol-requiring enzyme 1 (IRE1) signaling pathways [76]. Western blot result showed that the phosphorylation of PERK was up-regulated by MPEE treatment, which could release GRP78, phosphorylate eIF2α and increase CHOP to induce apoptosis [77]. Consistently, the phosphorylation of eIF2α and the levels of GPR78 and CHOP were up-regulated by MPEE treatment. Moreover, the RNA and protein levels of ATF6 were increased by MPEE treatment, which could enhance the expression of GPR78 and CHOP. CHOP could promote the expression of GADD34 and the up-regulated expression of GADD34 was observed upon MPEE treatment, which was involved in apoptosis [78]. The results indicated that MPEE induced apoptosis of HCC cells through ER stress signaling pathway. The various components of MPEE might be endowed the pleiotropic effects on the induction of cell cycle arrest and apoptosis through different signaling pathways.

Cisplatin is a well-known chemotherapeutic drug. It has been employed for treatment of numerous human cancers, such as testicular, ovarian, colorectal, bladder, lung and liver cancer. Cisplatin exerts anticancer effects via multiple mechanisms including its most prominent ability to cross-link with DNA to block transcription and replication, and induce mitochondria-dependent apoptosis. However, cisplatin can cause severe side effects, such as nephrotoxicity, cardiotoxicity and gastrointestinal toxicity [79, 80]. In our study, MPEE significantly suppressed the growth of tumor and greatly improved the survival of tumor mice without obvious side effect. In the future study, we will investigate the antitumor effect of MPEE on the metastatic tumor mouse model.

## Conclusion

MPEE suppressed the growth of HCC cells both in vitro and in vivo through induction of intrinsic- and ER stress-associated apoptosis. MPEE also inhibited the migration of HCC cells in vitro and improved the survival of tumor mice. These results indicate that MPEE may be a promising candidate for the treatment of HCC.

## Supplementary Information

**Additional file 1: Figure S1**. Statistical analysis for data of Western blot in Figs. 2F,4C and 4E.

**Additional file 2: Figure S2**. Statistical analysis for data of Western blot in Figs. 7C

**Additional file 3: Figure S3**. A total ion chromatogram from MPEE sample (ESI- and ESI +)

**Additional file 4: Table S1**. Main active ingredients identified under negative ESI mode (ESI-)by Liquid Chromatography Quadrupole Time-of-Flight Tandem Mass Spectrometry (LC-Q-TOF–MS) and their Contents in the MPEE

**Additional file 5: Table S2**. Main active ingredients identified under positive ESI mode (ESI +)by Liquid Chromatography Quadrupole Time-of-Flight Tandem Mass Spectrometry (LC-Q-TOF–MS) and their Contents in the MPEE.

## Data Availability

All the data used to support the findings of this study are available from the corresponding author upon reasonable request.

## Abbreviations

MPEE: Marchantia polymorpha ethanol extract
HCC: Hepatocellular carcinoma
ER: Endoplasmic reticulum
MTT: 3-(4,5-Dimethyl-2-thiazolyl)-2,5-diphenyl-2-*H*-tetrazolium bromide
DMSO: Dimethyl sulfoxide
PI: Propidium iodide
ΔψM: Mitochondrial membrane potential
ROS: Reactive oxygen species
qRT-PCR: Quantitative real time polymerase chain reaction
PARP: Poly (ADP-ribose) polymerase
MCM: Minichromosome maintenance family
CDKs: Cyclin-dependent kinases
DCFH-DA: Dichlorodihydrofluorescein diacetate
BCL-2: The B cell lymphoma 2 family
Bax: Bcl-2-associated X protein
p-PERK: Phosphorylated protein kinase-like ER kinase
GRP78/BiP: PERK releases glucose-regulated protein 78
eIF2α: Phosphorylates eukaryotic translation initiation factor 2 alpha
ATF6: Activating transcription factor 6
CHOP: C/EBP homologous protein
Gadd45a: DNA damage-inducible 45 alpha
UPR: The unfolded protein response
IRE1: Inositol-requiring enzyme 1
