# Supplementary figures and images for: Marchantia polymorpha L. ethanol extract induces apoptosis in hepatocellular carcinoma cells via intrinsic- and endoplasmic reticulum stress-associated pathways
Source: Chin Med. 2021 Sep 28;16:94. doi: 10.1186/s13020-021-00504-4 (PMC8477563; doi:10.1186/s13020-021-00504-4)

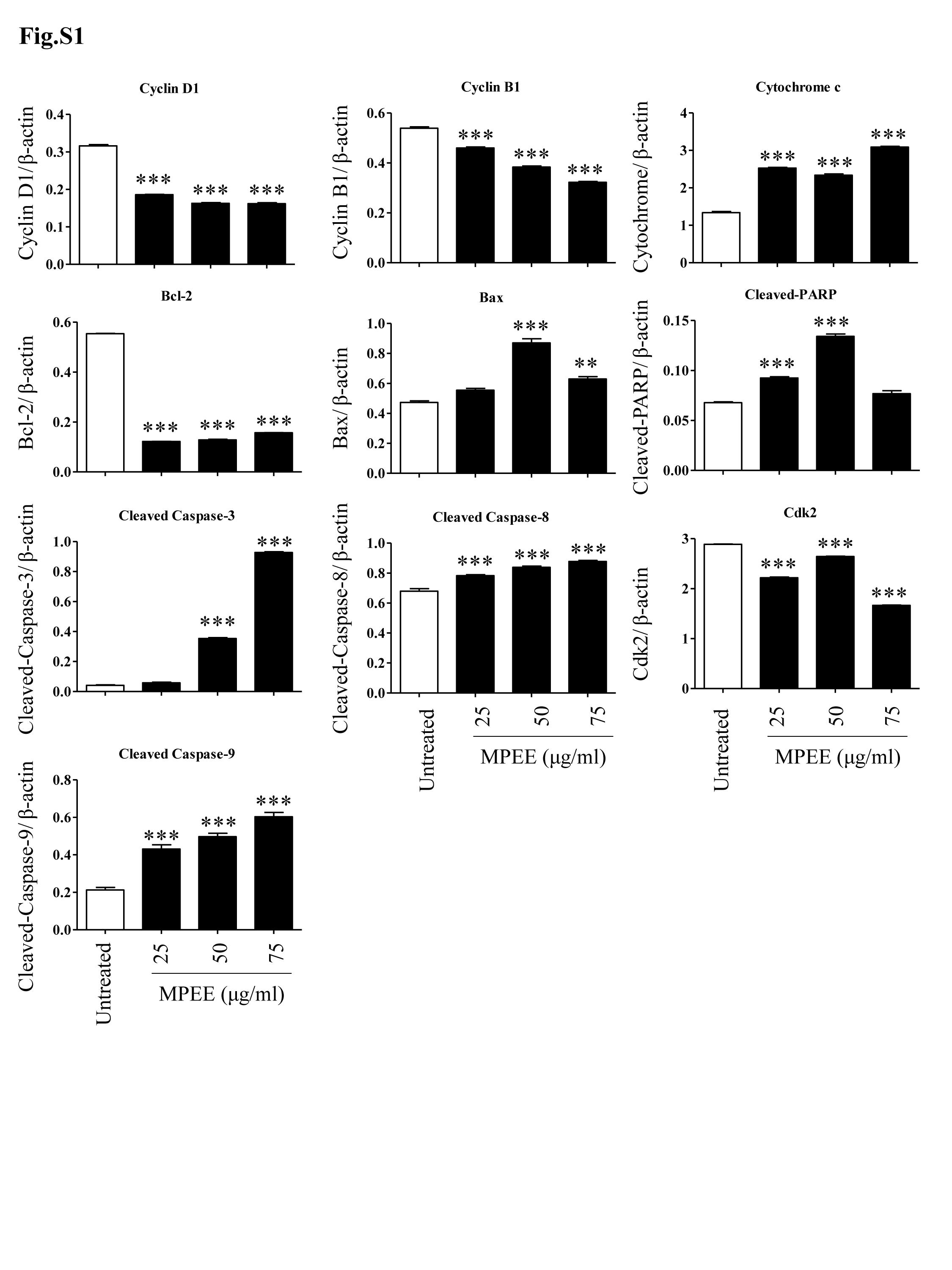

Supplement: Supplementary file 1 — Additional file 1: Figure S1. Statistical analysis for data of Western blot in Figs. 2F,4C and 4E. [file 13020_2021_504_MOESM1_ESM.tif]

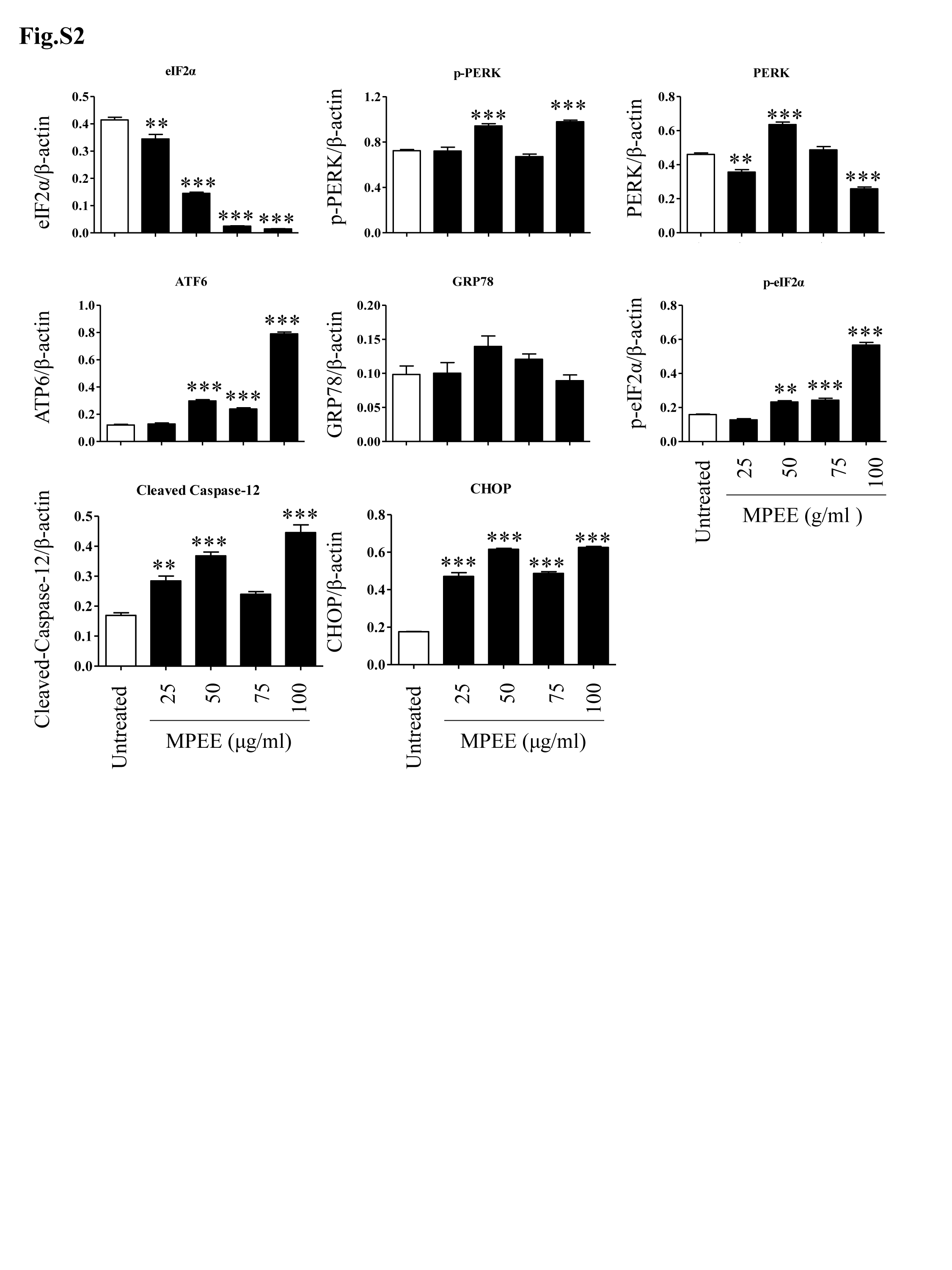

Supplement: Supplementary file 2 — Additional file 2: Figure S2. Statistical analysis for data of Western blot in Figs. 7C [file 13020_2021_504_MOESM2_ESM.tif]

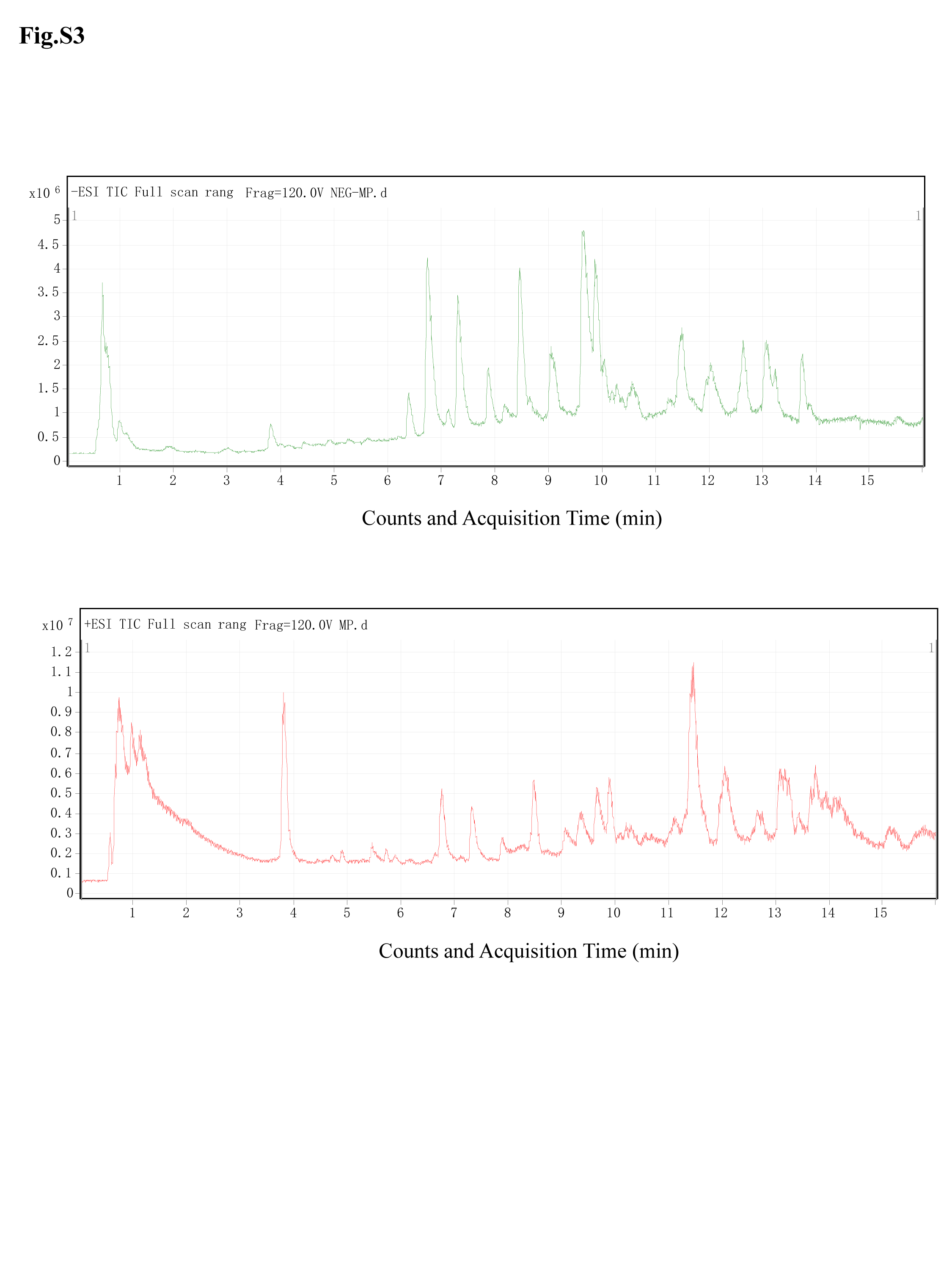

Supplement: Supplementary file 3 — Additional file 3: Figure S3. A total ion chromatogram from MPEE sample (ESI- and ESI +) [file 13020_2021_504_MOESM3_ESM.tif]
