# Supplementary material for: Marchantia polymorpha L. ethanol extract induces apoptosis in hepatocellular carcinoma cells via intrinsic- and endoplasmic reticulum stress-associated pathways
Source: Chin Med. 2021 Sep 28;16:94. doi: 10.1186/s13020-021-00504-4 (PMC8477563; doi:10.1186/s13020-021-00504-4)
Supplement: Supplementary file 4 — Additional file 4: Table S1. Main active ingredients identified under negative ESI mode (ESI-)by Liquid Chromatography Quadrupole Time-of-Flight Tandem Mass Spectrometry (LC-Q-TOF–MS) and their Contents in the MPEE [file 13020_2021_504_MOESM4_ESM.docx]

| **Table S1 Main active ingredients identified under negative ESI mode (ESI-)by Liquid Chromatography Quadrupole Time-of-Flight Tandem Mass Spectrometry (LC-Q-TOF-MS) and their Contents in the MPEE** | | | | | | | |
| --- | --- | --- | --- | --- | --- | --- | --- |
| Mass | RT（min） | PPM | molecular_weight | Name | class | [Area] MP | References |
| 138.0315 | 3.99 | 1 | 138.0316941 | 4-Hydroxybenzoic acid | Benzene and substituted derivatives | 274.3190329 | [28] |
| 226.0648 | 6.39 | 8 | 226.0629942 | 2-Methoxyxanthone | Benzopyrans | 1670.71502 | NO |
| 378.15 | 12.65 | 9 | 378.1467238 | Demethylcalabaxanthone | Benzopyrans | 334.4525293 | NO |
| 380.1658 | 13.25 | 9 | 380.1623739 | Garcinone A | Benzopyrans | 241.591866 | [29] |
| 500.1817 | 8.56 | 4 | 500.1835033 | Artonol C | Benzopyrans | 140.7493645 | NO |
| 159.0893 | 4.88 | 2 | 159.0895433 | Betonicine | Carboxylic acids and derivatives | 100.428042 | NO |
| 174.0165 | 1.03 | 0 | 174.0164379 | cis-Aconitic acid | Carboxylic acids and derivatives | 366.2657896 | [30] |
| 230.0942 | 7.13 | 17 | 230.0902716 | 1-(gamma-Glutamylamino)cyclopropanecarboxylic acid | Carboxylic acids and derivatives | 436.9055242 | NO |
| 260.1057 | 7.88 | 19 | 260.1008363 | Oxypinnatanine | Carboxylic acids and derivatives | 926.5374391 | NO |
| 520.166 | 9.89 | 15 | 520.1580763 | 3,4,5-trihydroxy-6-[(2-{9-[(3-methylbut-2-enoyl)oxy]-2-oxo-2H,8H,9H-furo[2,3-h]chromen-8-yl}propan-2-yl)oxy]oxane-2-carboxylic acid | Coumarins and derivatives | 206.300716 | NO |
| 600.1761 | 9.22 | 12 | 600.169035 | 3,4,5-trihydroxy-6-{[7-oxo-2-(2-{[3,4,5-trihydroxy-6-(hydroxymethyl)oxan-2-yl]oxy}propan-2-yl)-2H,3H,7H-furo[3,2-g]chromen-9-yl]oxy}oxane-2-carboxylic acid | Coumarins and derivatives | 144.0568912 | NO |
| 602.2148 | 8.33 | 14 | 602.2062275 | Tipranavir | Diarylheptanoids | 214.8662126 | [31] |
| 130.0269 | 1.03 | 2 | 130.0266087 | Itaconic acid | Fatty Acyls | 132.9115005 | [32] |
| 256.2393 | 13.74 | 4 | 256.2402303 | Butyl dodecanoate | Fatty Acyls | 104.7360149 | NO |
| 258.2194 | 12.02 | 0 | 258.2194948 | 10-Hydroxymyristic acid methyl ester | Fatty Acyls | 358.4985544 | NO |
| 278.2246 | 12.66 | 0 | 278.2245802 | Gamma-Linolenic acid | Fatty Acyls | 296.0312009 | [33] |
| 280.2387 | 13.24 | 5 | 280.2402303 | Linoelaidic acid | Fatty Acyls | 121.6330015 | [34] |
| 288.2305 | 11.51 | 2 | 288.2300595 | (S)-10,16-Dihydroxyhexadecanoic acid | Fatty Acyls | 1499.596406 | NO |
| 296.2328 | 10.87 | 8 | 296.2351449 | 9,10-Epoxyoctadecenoic acid | Fatty Acyls | 180.1936473 | [35] |
| 302.2207 | 12.56 | 13 | 302.2245802 | Eicosapentaenoic acid | Fatty Acyls | 633.3057803 | [36] |
| 312.2305 | 11.1 | 1 | 312.2300595 | (Â±)-(E)-13-Hydroxy-10-oxo-11-octadecenoic acid | Fatty Acyls | 199.3402798 | NO |
| 286.0487 | 6.74 | 3 | 286.047738 | 3,5,7-trihydroxy-2-(3-hydroxyphenyl)-4H-chromen-4-one | Flavonoids | 10461.23368 | NO |
| 300.0637 | 7.48 | 1 | 300.0633881 | 5,7-dihydroxy-2-(3-hydroxy-5-methoxyphenyl)-4H-chromen-4-one | Flavonoids | 239.368609 | NO |
| 342.1161 | 0.67 | 17 | 342.1103383 | Zapotin | Flavonoids | 856.6699653 | [37] |
| 436.1325 | 10.19 | 10 | 436.136947 | 2-(3-hydroxyphenyl)-6-[3,4,5-trihydroxy-6-(hydroxymethyl)oxan-2-yl]-3,4-dihydro-2H-1-benzopyran-3,5,7-triol | Flavonoids | 616.5772164 | NO |
| 450.1122 | 7.06 | 9 | 450.1162115 | 6-{[3,5-dihydroxy-2-(3-hydroxyphenyl)-3,4-dihydro-2H-1-benzopyran-7-yl]oxy}-3,4,5-trihydroxyoxane-2-carboxylic acid | Flavonoids | 217.2152046 | NO |
| 478.1405 | 9.63 | 15 | 478.1475117 | 6-{[2-(4-ethyl-3-hydroxyphenyl)-3,5-dihydroxy-3,4-dihydro-2H-1-benzopyran-7-yl]oxy}-3,4,5-trihydroxyoxane-2-carboxylic acid | Flavonoids | 113.3577818 | NO |
| 492.1352 | 8.46 | 17 | 492.1267762 | 2-(2,5-dihydroxy-4-methoxyphenyl)-5-hydroxy-7-methoxy-6-[3,4,5-trihydroxy-6-(hydroxymethyl)oxan-2-yl]-4H-chromen-4-one | Flavonoids | 121.55888 | NO |
| 516.205 | 5.65 | 11 | 516.1995472 | 3,4,5-trihydroxy-6-{4-[1-hydroxy-3-(5-methoxy-2,2-dimethyl-2H-chromen-6-yl)propyl]phenoxy}oxane-2-carboxylic acid | Flavonoids | 179.4442827 | NO |
| 418.1658 | 9.03 | 7 | 418.1627678 | Lirioresinol A | Furanoid lignans | 3348.088558 | NO |
| 194.042 | 0.73 | 10 | 194.0401503 | Bis(2-furanylmethyl) sulfide | Heteroaromatic compounds | 156.2388187 | NO |
| 90.0316 | 0.65 | 1 | 90.03169406 | Hydroxypropionic acid | Hydroxy acids and derivatives | 312.6767177 | NO |
| 134.0213 | 0.83 | 2 | 134.0215233 | L-Malic acid | Hydroxy acids and derivatives | 247.0811262 | [38] |
| 152.0338 | 1.01 | 2 | 152.0334254 | Xanthine | Imidazopyrimidines | 209.8601005 | [39] |
| 145.0521 | 5.94 | 5 | 145.0527639 | 1H-Indole-3-carboxaldehyde | Indoles and derivatives | 117.777131 | NO |
| 270.0564 | 7.31 | 13 | 270.0528234 | 5,7-dihydroxy-3-(3-hydroxyphenyl)-4H-chromen-4-one | Isoflavonoids | 5855.379242 | NO |
| 350.1137 | 8.35 | 5 | 350.1154237 | 4'-O-Methylkanzonol W | Isoflavonoids | 111.7531094 | NO |
| 518.1414 | 9.13 | 2 | 518.1424263 | 6-{[3-(3,4-dimethoxyphenyl)-7-methoxy-8-methyl-4-oxo-4H-chromen-5-yl]oxy}-3,4,5-trihydroxyoxane-2-carboxylic acid | Isoflavonoids | 138.6518424 | NO |
| 219.0896 | 4.43 | 0 | 219.0895433 | Nigellimine N-oxide | Isoquinolines and derivatives | 132.4019636 | NO |
| 704.1677 | 11.94 | 9 | 704.1741204 | Occidentoside | Lignan glycosides | 128.5096145 | NO |
| 258.089 | 7.84 | 1 | 258.0892089 | 3-phenyl-1-(2,4,6-trihydroxyphenyl)propan-1-one | Linear 1,3-diarylpropanoids | 117.9793158 | NO |
| 288.096 | 1.95 | 13 | 288.0997736 | 3-(3,4-dihydroxy-2-methoxyphenyl)-1-(3-hydroxyphenyl)propan-1-one | Linear 1,3-diarylpropanoids | 128.9543435 | NO |
| 356.1654 | 13.75 | 8 | 356.1623739 | 1-(3,4-dihydroxyphenyl)-3-[4-hydroxy-2-methoxy-3-(3-methylbut-2-en-1-yl)phenyl]propan-1-one | Linear 1,3-diarylpropanoids | 364.0840561 | NO |
| 294.2198 | 11.49 | 1 | 294.2194948 | 10-Oxo-11-octadecen-13-olide | Macrolides and analogues | 725.318121 | NO |
| 250.1928 | 11.67 | 2 | 250.1932801 | Norambreinolide | Naphthofurans | 245.0984729 | NO |
| 334.1212 | 8.79 | 14 | 334.1164863 | Cappariloside A | Organooxygen compounds | 179.604944 | NO |
| 382.1815 | 13.9 | 6 | 382.1838972 | 1,2,10-Trihydroxydihydro-trans-linalyl oxide 7-O-beta-D-glucopyranoside | Organooxygen compounds | 140.3263675 | NO |
| 402.1503 | 12.57 | 6 | 402.1525971 | Benzyl beta-primeveroside | Organooxygen compounds | 170.7992316 | NO |
| 438.1509 | 9.67 | 4 | 438.1525971 | 7-Hydroxy-5-(4-hydroxy-2-oxopentyl)-2-methylchromone 7-glucoside | Organooxygen compounds | 3065.943691 | NO |
| 484.1934 | 9.87 | 18 | 484.1845659 | Nb-trans-p-Coumaroylserotonin glucoside | Organooxygen compounds | 7380.250306 | NO |
| 154.0616 | 3.01 | 9 | 154.0629942 | Hydroxytyrosol | Phenols | 322.8352446 | [40] |
| 266.1877 | 9.39 | 2 | 266.1881947 | 4-Hydroxy-3-methoxy-2,10-bisaboladien-9-one | Prenol lipids | 143.0304441 | [41] |
| 304.2399 | 13.11 | 1 | 304.2402303 | ent-17-Hydroxy-16beta-kauran-19-al | Prenol lipids | 360.6597202 | NO |
| 310.2143 | 10.5 | 0 | 310.2144094 | Auxin b | Prenol lipids | 580.1218542 | [42] |
| 340.2378 | 13.11 | 7 | 340.2402303 | Plastoquinone 3 | Prenol lipids | 104.5408153 | [43] |
| 424.1683 | 10.58 | 12 | 424.1733325 | Taraxinic acid glucosyl ester | Prenol lipids | 407.0302889 | NO |
| 456.3633 | 13.06 | 6 | 456.3603454 | Boswellic acid | Prenol lipids | 120.3131731 | [44] |
| 472.3556 | 11.3 | 1 | 472.35526 | Lucidumol A | Prenol lipids | 288.4785688 | [45] |
| 482.1742 | 10.66 | 7 | 482.1707457 | Melleolide D | Prenol lipids | 450.6560335 | [46] |
| 242.0901 | 1.96 | 1 | 242.0902716 | Thymidine | Pyrimidine nucleosides | 147.4672565 | [47] |
| 426.1859 | 10.05 | 7 | 426.1889826 | 11,13-Dihydrotaraxinic acid glucosyl ester | Saccharolipids | 1510.65681 | NO |
| 444.3581 | 12.05 | 5 | 444.3603454 | (3beta,5alpha,6beta,22E,24R)-23-Methylergosta-7,22-diene-3,5,6-triol | Steroids and steroid derivatives | 408.578691 | NO |
| 544.189 | 9.75 | 10 | 544.1944619 | Physalin E | Steroids and steroid derivatives | 112.6953451 | [48] |
| 214.0989 | 7.86 | 2 | 214.0993797 | (+)-(1R,2R)-1,2-Diphenylethane-1,2-diol | Stilbenes | 353.5110697 | NO |
| 166.0479 | 0.67 | 4 | 166.0486065 | S-Propyl 1-propanesulfinothioate | Thiosulfinic acid esters | 314.0497119 | NO |
| 199.0401 | 3.82 | 0 | 199.04 | DL-o-Chlorophenylalanine |  | 560 | NO |
| 540.1474 | 0.67 | 16 | 540.1559846 | N,N'-Bis(g-glutamyl)-3,3'-(1,2-propylenedithio)dialanine |  | 115.9675573 | NO |
