# Supplementary material for: Marchantia polymorpha L. ethanol extract induces apoptosis in hepatocellular carcinoma cells via intrinsic- and endoplasmic reticulum stress-associated pathways
Source: Chin Med. 2021 Sep 28;16:94. doi: 10.1186/s13020-021-00504-4 (PMC8477563; doi:10.1186/s13020-021-00504-4)
Supplement: Supplementary file 5 — Additional file 5: Table S2. Main active ingredients identified under positive ESI mode (ESI +)by Liquid Chromatography Quadrupole Time-of-Flight Tandem Mass Spectrometry (LC-Q-TOF–MS) and their Contents in the MPEE. [file 13020_2021_504_MOESM5_ESM.docx]

| **Table S2 Main active ingredients identified under positive ESI mode (ESI+)by Liquid Chromatography Quadrupole Time-of-Flight Tandem Mass Spectrometry (LC-Q-TOF-MS) and their Contents in the MPEE** | | | | | | | |
| --- | --- | --- | --- | --- | --- | --- | --- |
| Mass | RT（min） | PPM | molecular_weight | Name | class | [Area] MP | References |
| 117.0808 | 0.71 | 1 | 117.0789786 | Betaine | Carboxylic acids and derivatives | 90.99545144 | [49] |
| 131.094 | 1.24 | 1 | 131.0946287 | L-Isoleucine | Carboxylic acids and derivatives | 154.9457686 | [50] |
| 218.0198 | 0.66 | 1 | 218.0215233 | 4,8-dihydroxy-2H-furo[2,3-h]chromen-2-one | Coumarins and derivatives | 61.24215469 | NO |
| 286.0469 | 6.75 | 7 | 286.047738 | 3,5,7-trihydroxy-2-(3-hydroxyphenyl)-4H-chromen-4-one | Flavonoids | 406.5545672 | NO |
| 436.1339 | 10.21 | 10 | 436.136947 | 2-(3-hydroxyphenyl)-6-[3,4,5-trihydroxy-6-(hydroxymethyl)oxan-2-yl]-3,4-dihydro-2H-1-benzopyran-3,5,7-triol | Flavonoids | 79.31643869 | NO |
| 270.0536 | 7.32 | 7 | 270.0528234 | Genistein | Isoflavonoids | 395.3929433 | [51] |
| 158.023 | 0.88 | 1 | 158.0215233 | 2-Methylene-4-oxopentanedioic acid | Keto acids and derivatives | 53.97249024 | NO |
| 136.0711 | 1.16 | 1 | 136.0735589 | 1-Deoxy-D-ribitol | Organooxygen compounds | 85.14812206 | NO |
| 234.1622 | 9.16 | 9 | 234.1619799 | Epiacoronene | Organooxygen compounds | 50.04174698 | NO |
| 380.0739 | 0.67 | 1 | 380.0777176 | 4-Methoxybenzyl O-(2-sulfoglucoside) | Organooxygen compounds | 54.96040626 | NO |
| 438.1514 | 8.49 | 8 | 438.1525971 | 7-Hydroxy-5-(4-hydroxy-2-oxopentyl)-2-methylchromone 7-glucoside | Organooxygen compounds | 324.8225007 | NO |
| 204.1905 | 12.06 | 12 | 204.1878008 | beta-Patchoulene | Polycyclic hydrocarbons | 611.7856408 | NO |
| 414.2077 | 9.65 | 10 | 414.2042387 | Armillaripin | Prenol lipids | 100.1808101 | NO |
| 624.2647 | 13.27 | 13 | 624.2629354 | Kanokoside D | Prenol lipids | 130.0801958 | [52] |
| 233.1759 | 5.4 | 5 | 233.1779644 | Rotundine B | Pyridines and derivatives | 67.73588883 | NO |
| 384.3362 | 11.12 | 11 | 384.339216 | 7-Dehydrocholesterol | Steroids and steroid derivatives | 75.96735013 | [53] |
| 454.1456 | 9.08 | 9 | 454.1416384 | Ampelopsin D | Stilbenes | 152.9096204 | [54] |
| 136.0399 | 1.01 | 1 | 136.0380418 | 1-Pentanesulfenothioic acid | Sulfenyl compounds | 90.52461845 | NO |
| 534.2655 | 14.11 | 14 | 534.263091 | Pyrophaeophorbide a | Tetrapyrroles and derivatives | 160.1560452 | NO |
| 199.0419 | 3.84 | 4 | 199.04 | DL-o-Chlorophenylalanine |  | 560 | NO |
